# Pedigree analysis and epidemiological features of idiopathic congenital talipes equinovarus in the United Kingdom: a case-control study

**DOI:** 10.1186/1471-2474-8-62

**Published:** 2007-07-05

**Authors:** AH Cardy, S Barker, D Chesney, L Sharp, N Maffulli, Z Miedzybrodzka

**Affiliations:** 1 Department of Public Health, University of Aberdeen, Polwarth Building, Foresterhill, Aberdeen AB25 2ZD, UK; 2 Department of Orthopaedic Surgery, Aberdeen University School of Medicine, Polwarth Building, Foresterhill, Aberdeen AB25 2ZD, UK; 3 Freeman Hospital, Freeman Road, Newcastle upon Tyne, NE7 7DN, UK; 4 National Cancer Registry of Ireland, Elm Court, Boreenmanna Road, Cork, Ireland; 5 Department of Trauma and Orthopaedic Surgery, Keele University School of Medicine, Thornburrow Drive, Stoke on Trent ST4 7QB, UK; 6 Department of Medicine and Therapeutics, University of Aberdeen, Polwarth Building, Foresterhill, Aberdeen AB25 2ZD, UK

## Abstract

**Background:**

Congenital talipes equinovarus (CTEV) is a common developmental disorder of the foot, affecting between 1 and 4.5 per 1000 live births. The aetiology is not well elucidated. While both genetic and environmental factors are implicated, no specific genes have been identified and little is known about environmental risk factors.

**Methods:**

We conducted a case-control study of idiopathic congenital talipes equinovarus (ICTEV) in the United Kingdom. 194 cases and 60 controls were recruited. Pedigrees were obtained for 167 cases.

**Results:**

The rank of the index pregnancy, maternal education and caesarean delivery were significantly associated with ICTEV risk in a multivariate model. There were suggestions that maternal use of folic acid supplements in the three months before the pregnancy decreased ICTEV risk, and that parental smoking during the pregnancy increased risk, although the associations were not statistically significant. One quarter of pedigrees showed a family history of CTEV, and autosomal dominant inheritance was suggested in some of these.

**Conclusion:**

Uterine restriction did not appear to have a strong influence on ICTEV development in our study. Large population-based studies are needed to clarify the aetiology of this common developmental disorder.

## Background

Congenital talipes equinovarus (CTEV) is a common developmental disorder of the foot. It is defined as fixation of the foot in cavus, adductus, varus and equinus (i.e. inclined inwards, axially rotated outwards, and pointing downwards) with concomitant soft tissue abnormalities [1]. CTEV may be syndromic, when it is associated with other neuromuscular and neurological disorders, or idiopathic (ICTEV). Severity varies from cases that resolve with conservative treatment, to rigid, deformed feet requiring prolonged management and surgery throughout childhood and early adulthood, with persistent disability and discomfort into later life. The birth prevalence of ICTEV is between 1 and 4.5 per 1000 [2]. The mechanism by which ICTEV develops is unknown; mechanical, neurological, muscular, bony, connective tissue and vascular mechanisms have all been proposed [3]. The aetiology is not well elucidated. Whilst both genetic and environmental factors are implicated, no specific genes have been identified and little is known about environmental risk factors [4].

The UK Talipes Study was designed to investigate the aetiology and describe patterns of management of ICTEV in the UK. In this article we report the epidemiological findings and describe pedigree analyses of families affected by ICTEV.

## Methods

Three groups of children with ICTEV, and their parents, were recruited between 1^st ^July 1993 and 1^st ^July 1997. All children had been diagnosed with ICTEV by a treating orthopaedic surgeon. The source of the first series was the Scottish Talipes Register, which was established in 1993 and included all hospitals involved in CTEV management [5]. The source of the second series was the North of England Talipes Register, which was set up in 1970 in the Doncaster and Montague Royal Infirmary and included children treated for CTEV at that hospital. The third series comprised children whose CTEV had been managed in Great Ormond Street Hospital for Sick Children, London, since 1982. Great Ormond Street effectively operated as a tertiary referral centre for South Eastern England.

Families were approached by letter and invited to participate by attending their local hospital for orthopaedic assessment and interview and by completing questionnaires. The orthopaedic assessment comprised notes review and clinical examination in order to confirm non-syndromic status. Family history was recorded on a pedigree for each family that attended for interview. Each family was approached only once. The questionnaires included sections on the nature of the ICTEV (laterality, details of operations, etc), pregnancy and birth of the affected (index) child, maternal reproductive history, parental smoking and alcohol use, parental education and ethnicity. Families were given the option of completing the questionnaires without attending interview. We recruited unaffected cousins as controls. These were sex and age-matched to cases where possible. The questionnaires were sent by the index family to the potential control family, and returned directly to the investigators.

Analyses were conducted using Stata 8 [6]. We carried out univariate analysis by computing unmatched odds ratios (OR) with 95% confidence intervals (CI) for each epidemiological variable, using logistic regression, adjusting for the matching variables of year of birth and sex. Analysis of parental smoking was conducted for all subjects combined and after stratification by the sex of the index child, since there are reports of sex differences in the literature [7,8]. The Mann-Whitney non-parametric test was used for comparison of birth weight. A multivariate regression model was built that included the matching variables and any factors for which the likelihood ratio test in the univariate analysis had a p value of <0.1. In addition, for cases, chi-squared tests were used to explore associations between the epidemiological variables and the child's sex and the laterality of the affected foot/feet. From the pedigrees, the total number of affected and unaffected 1st and 2nd degree relatives was calculated and the ratio of affected to total relatives of each degree was calculated.

The study was approved by the Grampian Research Ethics Committee.

## Results

458 ICTEV subjects were eligible for the study and 194 participated by completing the questionnaires (response rate 42%); 167 also attended for interview. Cousin controls were recruited for 60 (31%) participants. The characteristics of the cases and controls are summarized in Table 1. Participants and non-participants from the Scottish Talipes Register were compared in terms of sex, laterality and period of birth and there were no significant differences (data not shown).

**Table 1:** Characteristics of cases and controls

Characteristic		Case^1^		Control^1^				
		n	(%)	n	(%)	p	OR^2^	(95% CI)
Sex of index child^3^	male	130	(67.0)	38	(63.3)	-	1.0	(ref)
	female	64	(33.0)	22	(36.7)	0.60	0.47	(0.47–1.56)
Affected foot male	left	26	(25.0)	-	-	-	-	-
	right	28	(26.9)	-	-	-	-	-
	both	50	(48.1)	-	-	-	-	-
Affected foot female	left	6	(12.8)	-	-	-	-	-
	right	15	(31.9)	-	-	-	-	-
	both	26	(55.3)	-	-	-	-	-
Year of birth	1963–1979	14	(7.2)	4	(6.7)	-		
	1980–1989	32	(16.5)	15	(25.0)	-		
	1990–1994	61	(31.4)	25	(41.7)	-		
	1995–1999	87	(44.8)	16	(26.7)	-		
Multiple pregnancy	yes	10	(5.1)	0	(0.0)	0.07	3.24^4^	-
	no	185	(94.9)	60	(100)			
Type of delivery	Cephalic	157	(80.7)	56	(93.3)	-	1.00	(ref)
	Breech	4	(2.1)	1	(1.7)	0.71	1.52	(0.16–14.19)
	Caesarean	33	(17.2)	3	(5.0)	0.04	3.71	(1.09–12.62)
Birth weight	mean	3622 g	3397 g	0.10^5^		-		
	range	1247–4564 g	1531–4508 g	-	-	-		
Stillbirth^6^		5	(2.6)	1	(1.7)		1.77	(0.20–15.81)
Miscarriage^6^		50	(25.8)	15	(25.4)		1.04	(0.53–2.03)
Abortion^6^		33	(17.2)	9	(15)		1.14	(0.51–2.56)
Age of mother at birth of index child	≤19	8	(4.2)	1	(1.7)	0.25	3.56	(0.41–31.06)
	20–24	39	(20.2)	12	(20.0)	0.53	1.25	(0.55–2.83)
	25–29	64	(33.2)	23	(38.3)	-	1.00	(ref)
	30–34	53	(27.5)	16	(26.7)	0.74	1.14	(0.54–2.39)
	35–39	23	(11.9)	7	(11.7)	0.79	1.14	(0.43–3.03)
	≥40	6	(3.1)	1	(1.7)	0.48	2.19	(0.25–19.35)
Age of father at birth of index child	≤19	2	(1.1)	1	(2.3)	0.62	0.53	(0.05–6.27)
	20–24	20	(11.4)	5	(11.4)	0.87	1.10	(0.36–3.40)
	25–29	43	(24.4)	7	(15.9)	0.34	1.16	(0.61–4.21)
	30–34	63	(35.8)	17	(38.6)	-	1.00	(ref)
	35–39	31	(17.6)	10	(22.7)	0.60	0.78	(0.31–1.94)
	≥40	17	(9.7)	4	(9.1)	0.82	1.15	(0.34–3.91)
Rank of index pregnancy	1	81	(41.8)	32	(53.3)	-	1.00	(ref)
	2	66	(34.0)	22	(36.7)	0.61	1.18	(0.63–2.23)
	3	27	(13.9)	4	(6.7)	0.10	2.60	(0.84–8.05)
	4+	20	(10.3)	2	(3.3)	0.09	3.67	(0.81–16.74)
Total gravidity of mother	1	36	(18.5)	8	(13.3)	-	1.00	(ref)
	2	68	(34.9)	29	(48.3)	0.20	0.56	(0.23–1.35)
	3	48	(24.6)	13	(21.7)	0.72	0.83	(0.31–2.23)
	4+	43	(22.1)	10	(16.7)	0.90	1.07	(0.37–3.05)
Total parity of mother	1	52	(26.8)	14	(23.3)	-	1.00	(ref)
	2	83	(42.8)	33	(55.0)	0.35	0.71	(0.34–1.45)
	3	38	(19.6)	10	(16.7)	0.88	1.08	(0.43–2.71)
	4+	21	(10.8)	3	(5.0)	0.28	2.15	(0.54–8.47)
Maternal use of folic acid in 3 months before or during first trimester of pregnancy with index child	no	144	(74.2)	46	(76.7)	-	1.00	(ref)
	yes	50	(25.8)	14	(23.3)	0.67	0.86	(0.43–1.73)
Maternal smoking during pregnancy with index child	no	129	(66.5)	44	(73.3)	-	1.00	(ref)
	yes	65	(33.5)	16	(26.7)	0.34	1.37	(0.72–2.62)
Paternal smoking during pregnancy with index child	no	101	(58.7)	30	(66.7)	-	1.00	(ref)
	yes	71	(41.3)	15	(33.3)	0.29	1.46	(0.72–2.95)
Maternal alcohol consumption during pregnancy with index child	no	127	(65.5)	34	(56.7)	-	1.00	(ref)
	yes	67	(34.5)	26	(43.3)	0.21	0.69	(0.38–1.24)
Maternal education or training beyond secondary school	no	70	(37.6)	30	(52.6)	-	1.00	(ref)
	yes	116	(62.4)	27	(47.4)	0.09	1.71	(0.93–3.16)
Paternal education or training beyond secondary school	no	52	(31.3)	12	(27.3)	-	1.00	(ref)
	yes	114	(68.7)	32	(72.7)	0.48	0.76	(0.35–1.62)

^1 ^Denominator varies according to available data

^2 ^Adjusted for year of birth and sex of index child

^3 ^Index child is the child with ICTEV, or cousin control

^4 ^χ^2 ^test

^5 ^Mann-Whitney U test

^6 ^At some time in the reproductive history of the mother

The ratio of males to females was 2.1:1. Fifty one percent had bilateral ICTEV (48% of males; 55% of females). Of the unilateral cases, more had right foot involvement than left (57% right; 43% left). Females who were affected unilaterally were more than twice as likely to be affected on the right than the left, whereas in males left and right sides were equally affected (females 29% left, 71% right; males 48% left, 52% right); the differences were not statistically significant. Three quarters of cases were born in 1990 or later.

There were ten twin pregnancies amongst the cases (5%), nine of which were reported to be non-identical; the zygosity was unknown for the tenth. None of the non-index twins had CTEV. There were no multiple pregnancies amongst controls. Eighty one percent of ICTEV deliveries were vaginal with cephalic presentation, 2% were vaginal with breech presentation and 17% were caesarean. In controls, the corresponding figures were 93%, 2% and 5%. Odds ratios, adjusted for sex and year of birth, were significantly raised for caesarean section (Table 1). The increased risk associated with caesarean section was not accounted for by maternal age nor the rank of the index pregnancy, as inclusion of these variables in the model had little impact on the risk estimate. The association persisted when the analysis was restricted to singletons (age and sex adjusted OR 3.01, 95% CI 0.87–10.38), and in the final multivariate model (see below). The mean birth weight of cases was greater than that of controls (3622 g and 3397 g respectively), but the difference was not statistically significant.

At some time in her reproductive history, 26% (50/192) of case and 25% (15/59) of control mothers had had at least one miscarriage; 3% (5/191) of case and 2% (1/60) of control mothers had had a stillbirth and 17% (33/192) of case and 15% (9/60) of control mothers had had at least one abortion.

The median age of mothers at the birth of the index child was 28 years for both cases and controls (case range 15–43, control range 18–40). The median age of fathers was 31 years for cases (range 17–58) and 30 years for controls (range 19–50). The median age of the mother at the birth of her first child was 24 years for cases (range 15–38) and 25 years for controls (range 16–38).

The index pregnancy was the first for 42% of case and 53% of control mothers. The risk of ICTEV increased significantly with the rank of the pregnancy (p for trend = 0.03). Adjusting for maternal age made minimal difference to the risk estimates. Further adjustment in the final multivariate model (see below) increased the risk estimates slightly. The mean parity was 2.2 for case (range 1–6) and 2.1 for control (range 1–6) mothers.

Around one quarter of mothers (26% cases; 23% controls) reported using nutritional supplements containing folic acid in the three months before the index pregnancy or in the first trimester. Use increased with the year of birth of the child and was 5% for births 1963–1979, 13% for 1980–1989, 23% for 1990–1994 and 28% for 1995–1999. The increase over time was statistically significant (p for trend <0.001). Use of these supplements was associated with a modest, but non-significant, reduced risk of ICTEV. When the analysis was restricted to folic acid taken in the three months prior to the pregnancy the effect was more pronounced (OR 0.55, 95% CI 0.23–1.29), but remained non-significant.

More case than control parents reported smoking during the index pregnancy (mothers: 34% cases, 27% controls; fathers: 41% cases, 33% controls); the differences were not significant. Whilst the association with maternal smoking was stronger in female than male children (male OR 1.16 [95% CI 0.53–2.55]; female OR 2.28 [95% CI 0.68–7.66]), the risk estimates were not statistically significant, nor was a formal test of interaction between sex and maternal smoking significant (p = 0.41). Fewer case than control mothers reported drinking alcohol during the index pregnancy (35% cases, 43% controls), but the differences were not significant.

More case than control mothers had further education or training (cases 62% [116/186]; controls 47% [27/57]), although the difference was not statistically significant. Fathers of cases and controls had similar levels of further education.

Table 2 shows the results of multivariate modeling, with mutually adjusted odds ratios and 95% confidence intervals. The final model included the matching variables (sex and year of birth), rank of the index pregnancy (1, 2, 3, 4+), type of birth (normal, breech, caesarean), and mother's further education or training (any/none). The model could not be fitted with twin status, as there were no twins amongst controls. There was a statistically significant trend of increasing risk with increasing pregnancy rank. Mothers for whom the index pregnancy was at least the fourth had over three and a half times the risk of an ICTEV child than a mother for whom the pregnancy was the first. There was a four-fold risk associated with caesarean compared with normal vaginal delivery. Risk of an ICTEV birth was increased by 75% among mothers with further education, although the risk estimate was not statistically significant. Risk estimates for models that included and excluded twins were similar.

**Table 2:** Final regression model from the case-control analysis

Risk factor		p	OR^1^	(95% CI)
*Rank of index pregnancy*	1	ref	1.00	(ref)
	2	0.38	1.35	(0.69–2.65)
	3	0.08	2.81	(0.87–9.03)
	4+	0.12	3.49	(0.73–16.80)
				*p for trend 0.03*
*Type of birth*	Normal	ref	1.00	(ref)
	Breech	0.61	1.83	(0.19–18.13)
	Caesarean	0.02	5.55	(1.27–24.27)
*Maternal further education*	None	ref	1.00	(ref)
	Some	0.07	1.80	(0.95–3.40)

^1 ^Adjusted for year of birth and sex of index child

In the case-only analysis there were no statistically significant associations between sex and laterality (unilateral/bilateral), nor between the sex of the case and multiple birth, type of birth, rank of the pregnancy, parental smoking, maternal alcohol use or maternal education. In addition, there were no associations between these variables and laterality (right only, left only, bilateral). For maternal use of folic acid, there were significantly fewer females than expected in the group whose mothers used supplements (χ^2^_1 _7.53, p = 0.01) but the test for interaction between sex and folic acid use was not significant (p = 0.18). Folic acid use was not associated with laterality.

A pedigree was available for 167 families. Families with and without pedigrees were compared for the variables in Table 1; there were no significant differences (data not shown). The sex ratio of the cases for whom a pedigree was obtained was 1.98:1 (111 [66.5%] male; 56 [33.5%] female), very similar to the main study. Almost one quarter (40/167, 24%) of families reported a family history of CTEV; 16.2% (27/167) had a 1^st^–3^rd ^degree family history, 7.8% (13/167) a 1^st ^degree family history and 4.2% (7/167) a 2^nd ^degree family history; 10.8% (18/167) had a 1st *or *2^nd ^degree family history, and 1.2% (n = 2) a 1^st ^*and *2^nd ^degree family history. Of the 13 families with a 1^st ^degree family history, there were six child-parent pairs, six sib-pairs and one trio in which both a parent and a sib were affected. Six families had three or more cases of CTEV in the family (3.6%); the pedigrees of three of these suggested autosomal dominant inheritance whilst the mode of inheritance was uncertain in the remainder.

Male index cases were more likely to have a family history than female index cases; 18.9% of males (n = 20) and 12.5% of females (n = 7) had a 1^st^–3^rd ^degree family history. Eight percent of males (n = 9) and 7.1% of females (n = 4) had a 1st degree family history, and 5.4% of males (n = 6) and 1.8% of females (n = 1) had a 2^nd ^degree family history. None of these differences reached statistical significance (data not shown).

Of the 167 index cases, 2.7% of 1^st ^degree relatives (14/528; 95% CI 1.46–4.41) and 0.5% of 2^nd ^degree relatives (7/1473; 95% CI 0.19–0.98) were reported to have CTEV. The absolute risk of any 1^st ^*or *2^nd ^degree relative being affected was 1.1% (21/2001; 95% CI 0.65–1.60). Slightly more relatives of male (1.2%, 16/1341; 95% CI 0.68–1.93) than female (0.8%, 5/660; 95% CI 0.25–1.76) index cases had ICTEV. The risk to each male relative, regardless of the sex of the case, was 1.2% (12/1017; 95% CI 0.61–2.05) and for each female relative was 0.9% (9/984; 95% CI 0.42–1.73). When the case was male the risk of CTEV was higher for male (1.5%, 10/675; 95% CI 0.71–2.71) than female (0.9%; 6/666; 95% CI 0.33–1.95) relatives. When the index case was female the risk of CTEV was slightly higher for female relatives (risk to males 0.6%; 2/342; 95% CI 0.07–2.10; risk to females 0.9%, 3/318; 95% CI 0.19–2.73). None of these differences reached statistical significance (data not shown).

Cross-tabulations between 1^st^–3^rd ^degree family history and the variables sex, laterality, maternal alcohol and maternal smoking showed no associations. There was a significant association between family history and paternal smoking (χ^2^_1 _= 4.61, p = 0.03), but not with maternal smoking (χ^2^_1 _= 0.51, p = 0.48).

## Discussion

The sex ratio in our study and the predominance of bilateral disease mirrors the pattern seen in other studies of children with ICTEV [9-19]. Of the unilateral cases, the slight predominance of right over left-sided ICTEV is in line with most reported series [2,10,12,16,19]. This, together with comparison of participants and non-participants of the Scottish part of the study, suggests that our series is not severely biased and that our results are likely to be generalisable, despite the relatively low participation rate.

Five percent of cases but no controls were twin births. The rate of twinning in our cases was significantly higher than the Scottish rate for 1996–2000 of 1.4% [20] (χ^2 ^19.99, p<0.001). If this rate held amongst controls, we would have expected one twin birth. Uterine restriction is the earliest known hypothesis proposed to account for CTEV, with references dating back to Hippocrates in the 5th century BC. Our finding of a higher twin birth rate in cases than controls could be considered to support this hypothesis. However, we also found a positive association between risk of ICTEV and the rank of the index pregnancy; if it is assumed that the primagravid uterus is more restrictive than a multigravid uterus, uterine restriction is not a major factor in our series.

The rate of caesarean delivery was significantly higher in our cases than controls (17% vs. 5%). We did not have information on why the caesareans were performed. Twin births accounted for some of the excess, as 6/10 twin births were delivered by caesarean section, but the association remained when the analysis was restricted to singletons. The frequency of caesarean deliveries for singleton ICTEV pregnancies in our study (14%) is consistent with figures for the Scottish population, taking into account the range of years of birth covered by our study [21]. Thus the observed association may be due to the lower than expected frequency of caesarean births among controls.

Maternal education and birth rank were also significant in our final model, both being positively associated with the risk of ICTEV. Maternal education, which may be seen as an indicator of socioeconomic status, has been infrequently reported and findings are inconsistent with positive [7], inverse [19] and no association [18] reported. The studies vary in their categorisation of 'further education'. If our study participants were biased in terms of education we might expect mothers of controls to have higher education than mothers of cases, and this was not the case. We found a positive association between risk of CTEV and parity, in contrast to the findings of Moorthi et al (2005) [19]. Birth rank and parity have not generally been found to be associated with ICTEV risk [7,8,10,18,22]. The associations between maternal education and birth rank and risk of CTEV require further investigation in other studies.

Folate appears to play a role in some congenital malformations, particularly neural tube defects [23]. An association has been reported between CTEV and higher levels of maternal plasma homocysteine, which is inversely related to plasma folate [24,25]. Approximately one quarter of mothers in our study reported peri-conceptional use of folic acid. Maternal use of supplements containing folic acid in the three months before the index pregnancy, or in the first trimester, was associated with a modestly reduced risk of ICTEV, although this was not statistically significant. The effect was most pronounced for use in the period immediately before the pregnancy (OR 0.55, 95% CI 0.23–1.29). Rotation of the foot into the plantar (sole on the ground) position commences between nine and 13 weeks, and continues throughout gestation [26]. The association with folic acid supplement use may be a chance finding, or may suggest that the initiation of ICTEV is earlier than previously thought. That folic acid may play a role in the development of CTEV is also indicated by a small decrease in the birth prevalence of ICTEV in Texas, United States, after the introduction of folic acid fortification of grains, although the decrease was not statistically significant [19]. Further, a United Kingdom case-parent triad study has demonstrated an association between the risk of ICTEV and the C677T polymorphism in the methylenetetrahydrofolate reductase gene, a gene that is involved in folate metabolism [27]. Further investigation of these intriguing results is warranted, particularly in view of the potential implications for public health policy on peri-conceptional folic acid supplementation.

Maternal smoking has been implicated as a risk factor for CTEV [7,28-30]. In line with this, we found that more case than control mothers smoked during pregnancy (34% vs. 27%), although the difference was not statistically significant. The prevalence of maternal smoking in our study is consistent with Scottish survey data for 1993–1997 [31]. Skelly et al 2002 [7] observed a stronger effect of maternal smoking in girls than boys, and our data also suggested this pattern; however Alderman et al (1991) report a stronger effect in boys [8]. Honein et al (2000) demonstrated an interaction between maternal smoking and family history of ICTEV, with an odds ratio of 20.30 (7.90–51.17) for the combination of maternal smoking and family history [30]. We found a significant association between family history and paternal smoking, but not with maternal smoking. If family history is considered a marker of genetic involvement, this relationship may indicate an interaction between smoking and an as-yet unknown gene or genes, although it is unclear why the relationship should be with paternal rather than maternal smoking.

One quarter of children had a family history of CTEV, which corresponds with the upper limit of the estimates reported in two series in the USA [16,32], but is slightly lower than the estimate from a series of 120 children from Glasgow [12]. The families from whom we obtained a pedigree did not differ from those from whom we did not in terms of the variables in Table 1. We restricted our analysis of risk of CTEV to family members to 1^st^–2^nd ^degree relatives as this was considered the most reliable data.

The proportion of CTEV families reported to have a 1^st ^degree family history differs widely. Our frequency of 7.8% is similar to that of 7.2% reported by Honein et al [30], whilst Skelly et al (2002) report 9.2% [7], Cartlidge (1984) 12.5% [12] and Wynne-Davies (1972) 21.4% [33]. The absolute risk of CTEV in 1^st ^degree relatives in our study was 2.7%, within the range reported in the literature (2.1–3.6%) [10,16,30,33].

We found an increased risk of a family history of CTEV in male compared with female index cases, although the difference was not statistically significant. The highest risk of CTEV in our study was in male relatives of male probands, contrary to Wynne-Davis (1964) who found the highest risk in male relatives of female probands in a series of 635 UK cases [34]. We had insufficient affected families to perform a formal segregation analysis.

The heterogeneity between studies in reported risk factors for CTEV may be due to many factors including definitions of CTEV, whether non-idiopathic cases are included, differences in study design, and chance as some series are relatively small. Participation bias may also play a role, possibly with multi-case families or those with more severely affected children more likely to take part. Studies of family history of CTEV are likely to vary in the degree of evidence considered sufficient for a 'diagnosis' of CTEV in relatives. The aetiology of CTEV is undoubtedly complex and it is probable that both environmental and genetic factors are involved. The genetic influence itself is likely to be complex and may involve multiple genes or genetic pathways, themselves interacting with environmental factors.

## Conclusion

We have given an epidemiological picture of a large series of children with ICTEV in the UK. Autosomal dominant inheritance seems to be present in some of the families, although the mode of inheritance was unclear for the majority of multi-case families. Cesarean delivery, birth rank and maternal education were all significantly associated with the risk of ICTEV. Uterine restriction does not seem to play a major role in our series. We found significant associations between family history and paternal smoking, which may indicate an interaction between smoking and an as-yet unknown gene or genes. Studies of unbiased series from different geographical and ethnic groups are essential for elucidating both the genetic and epidemiological factors involved in the aetiology of this common development disorder.

## Abbreviations

CTEV – congenital talipes equinovarus

ICTEV – idiopathic congenital talipes equinovarus

OR – odds ratio

95%CI – 95% confidence interval

## Competing interests

The author(s) declare that they have no competing interests.

## Authors' contributions

AC conducted the analysis and drafted the paper. SB and DC conducted orthopaedic assessment of participants, collected the data and commented on the paper. LS contributed to study design, running the study, advised on analysis and contributed to paper writing. NM conceived the original study and commented on the paper. ZM contributed to study design, running the study and to paper writing. All authors read and approved the final manuscript.

## Pre-publication history

The pre-publication history for this paper can be accessed here:


